# Maternal Hypercholesterolemia May Involve in Preterm Birth

**DOI:** 10.3389/fcvm.2022.818202

**Published:** 2022-07-11

**Authors:** Jingfei Chen, Lan Hua, Fei Luo, Jianlin Chen

**Affiliations:** ^1^Department of Obstetrics and Gynecology, The Second Xiangya Hospital, Central South University, Changsha, China; ^2^Department of Cardiovascular Medicine, The Second Xiangya Hospital, Central South University, Changsha, China

**Keywords:** preterm birth, macrophage, cholesterol, low-density lipoprotein receptor, inflammation

## Abstract

Maternal hypercholesterolemia during pregnancy is associated with an increased risk of preterm birth which is defined as <37 weeks of complete gestation. However, the underlying mechanism for the association between hypercholesterolemia and preterm birth is not fully understood. Macrophage, as one of the largest cell types in the placenta, plays a very critical role in mediating inflammation and triggers labor initiation. Here, we hypothesize that macrophages can uptake maternal excessive cholesterol leading to its accumulation, resulting in a breach of the immune tolerance and precipitating labor.

## Introduction

The hydrophobic lipid, cholesterol (C27H46O), first isolated from human gallstones more than two centuries ago, plays a critical role in maintaining normal human physiology. Disruption in cholesterol metabolism can cause congenital human diseases (such as Familial hypercholesterolemia, Tangier disease, Schnyder corneal dystrophy) and acquired diseases (such as atherosclerosis, cardiovascular disease, and Alzheimer's disease) (1).

Cholesterol is a structural component and presents in every cellular membrane, which is also essential for embryonic and fetal development. Burgeoning evidence supports the role of cholesterol in parturition with very high level of cholesterol in pregnant women being associated with an increased risk of preterm birth (PTB) in comparison to women with moderate cholesterol level (2, 3). A meta-analysis involving 13,025 pregnant women found that maternal dyslipidemia during pregnancy, either the elevated total cholesterol or triglycerides, was associated with an increased risk of PTB (4). Besides, the cholesterol transporters such as ATP-binding cassette (ABC)-transporters, ABCA1 and ABCG1 are involved in parturition (5, 6) as evidenced by the study showing the association between abnormal expression of ABCA1 to the dysregulation of placental lipid metabolism and development of spontaneous PTB (7).

PTB is defined as delivery prior to 37 weeks gestational age. With an estimated global incidence of ~15 million per year, PTB constitutes the leading cause of neonatal morbidity and mortality worldwide (8), thereby putting a tremendous emotional and economic burden on society. Premature infants are also at risk of developing immediate complications like respiratory distress syndrome, sepsis, intraventricular hemorrhage, necrotizing enterocolitis, hypothermia, hypoglycemia, hyperbilirubinemia and long-term morbidity like retinopathy of prematurity, neurodevelopmental impairment, and cerebral palsy (8). However, pathophysiology of initiation of labor is poorly understood, the therapeutic strategies of prevention and treatment of PTB is limited. Cholesterol from pregnant woman with hypercholesteremia may get transported into placenta and accumulate, which triggers an inflammation response and results in PTB. Therefore, it is important to illustrate underlying mechanism of maternal dyslipidemia that can lead to premature delivery, with an aim of providing novel therapeutic target.

## Presentation Of The Hypothesis

Massive amounts of cholesterol are needed for growth. Fetuses have two sources of cholesterol, *de novo* synthesized cholesterol and exogenous cholesterol from maternal circulation *via* the placenta. Well-documented evidence has shown that fetus procure cholesterol from their mother (9). Newborns have a large amount of plant sterols, the content of which is about 40–50% of that in the mother (10). Since plant sterols are only obtained from the diet, their presence in fetal serum indicates their vertical transmission. Substantial cholesterol is also present in fetus who are unable to synthesize cholesterol due to genetic abnormalities (11, 12). A recent study also found that there was substantial uptake of cholesterol from mother (measured as difference in the arterial-venous concentrations) by the fetus using a 4-vessel sampling method (13). Transfer of maternal cholesterol to the embryo as well as the fetus was also confirmed in mice (14). Maternal cholesterol must cross trophoblast barrier of placenta to reach the fetal circulation (15), suggesting entry of maternal cholesterol through placenta.

The various cell types in the placental disc include trophoblasts, macrophages, connective tissue fibroblasts and vascular cells. It is unclear which cell type in the placenta contributes to uptake of cholesterol. In maternal circulation, the majority of cholesterol is in the form of LDL-cholesterol or HDL-cholesterol, which can be uptake by trophoblast of placenta *via* its low-density lipoprotein receptor (LDLR) and scavenger receptor class B type I (SR-B1), respectively (9). Macrophages take up native and modified (for example, oxidized) LDL-cholesterol *via* micropinocytosis, phagocytosis, or scavenger receptor-mediated pathways (including *via* SR-B1, lectin-like oxidized LDL receptor 1, and CD36) (16). Macrophages were reported to express multiple scavenger receptors for uptake of LDL, which promotes the cellular accumulation of cholesterol (17). Scavenger receptors constitute a heterogeneous family of receptors including CD36 and SR-B1. CD36 is a membrane glycoprotein that is expressed on various types of cells, which can bind to multiple ligands and mediate the endocytosis of LDL (18). SR-B1 can mediate cholesteryl esters selective uptake and the bi-directional flux of free cholesterol (19). Peroxisome proliferator–activated receptors (PPARs) and liver X receptors (LXRs) are members of the nuclear receptor superfamily of transcription factors that play a key role in regulating the expression of scavenger receptors of macrophages (20). PPARγ is required for placental development and regulates essential placental functions (21), which may also be a drug target for complicated pregnancy (22). LXR was also reported to be an important factor in early-pregnancy lipogenesis which is necessary to protect against abnormalities in fetoplacental lipid homeostasis (23). However, whether PPARγ and LXRs involve in the uptake of cholesterol by macrophages in the placenta needs more studies.

Besides, cholesterol can also transfer from one cell type to adjacent different cell types (24), raising a possibility that the excess cholesterol of trophoblast may be a result of cholesterol accumulation by adjacent macrophage. Down-regulation of lipids associated receptors of placenta such as LDLR, SR-B1 results in decreased uptake of cholesterol by placental from maternal circulation (5). The mRNA expression of lipoprotein receptors, including LDLR and very low-density lipoprotein receptor (VLDLR) was significantly increased in placenta from hypercholesterolemic women as comparison to the control group (25). A plausible explanation can be the uptake of LDL-cholesterol by placenta may lead to upregulation of lipoprotein receptors, which in turn, results in uptake of LDL-cholesterol in placenta macrophages.

A large body of literature shows the accumulated LDL-cholesterol in tissues can be modified to function as a ligand for macrophage pattern recognition receptors, including Toll-like receptors (TLRs), thereby directly triggering pro-inflammatory signaling pathways (26). Besides, accumulation of LDL-cholesterol in macrophages through endocytosis can also trigger TLR signaling (27, 28). Production of cytokines and chemokines may be amplified by increased TLR, amplifying the inflammatory process (27, 28).

**Figure 1:**
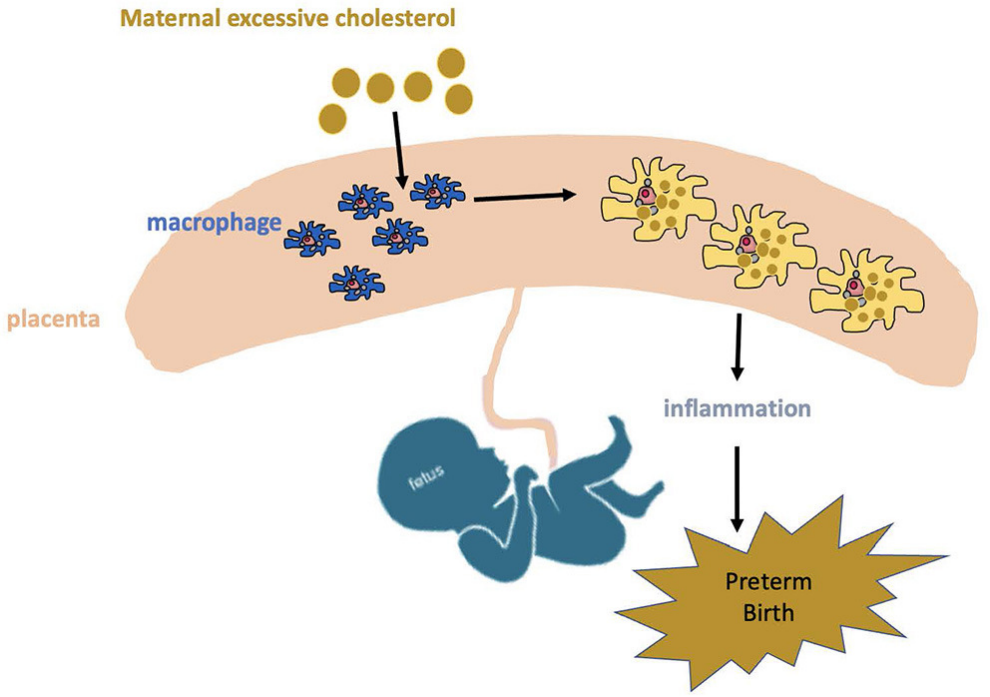
Potential mechanism of maternal hypercholesterolemia contributing to preterm birth. Excessive cholesterol from mother with hypercholesterolemia will be transported into placenta and accumulates in macrophages, which will increase inflammation response and consequently trigger preterm birth.

Macrophages are a major type of leukocytes in the placenta and play a critical role throughout pregnancy (29). These placental macrophages support a variety of processes essential for successful pregnancy such as remodeling of the uterine connective tissues and blood vessels, regulation of trophoblast implantation, immune-tolerance toward fetal antigens, immunomodulation of neighboring leukocytes and initiation of parturition (30). Besides, placental macrophages are an important component for suppression of maternal immunologic response to the allogenic placenta and fetus due to its decreased ability to present antigens to T cells during pregnancy. Near term, placental macrophage activity switched to inflammation state contributes to parturition through production of pro-inflammatory cytokines and prostaglandin E2 (31), which in turn break the immune tolerance and initiate parturition. Accumulation of cholesterol in macrophages can trigger pro-inflammatory signaling pathways (26). It is well-validated that pregnant women with hypercholesterolemia are associated with increased risk of PTB (2, 3, 32). Therefore, we hypothesize excessive cholesterol from the mother with hypercholesterolemia will be transported into the placenta and accumulate in macrophages, which will subsequently increase the inflammation, triggering PTB (Figure 1).

## Testing The Hypothesis

We will design some experiments to test this hypothesis. ①C57BL/6 male and female mice will be randomly given normal chow diet or 5% high cholesterol diet for 2–4 months (need to be optimized). Set up timed breeding and monitor the timing of parturition. At the endpoint of the experiment, mice will be sacrificed and blood, placenta will be collected. We will measure cholesterol levels in blood and placenta. We will isolate macrophage in placenta and measure lipids levels and the expression of cholesterol-uptake associated receptors including VLDLR, LDLR, CD36, and SR-B1. ②We will isolate macrophages from placenta by magnetic-activated cell sorting (MACS), then detect the cytokine, lipids and inflammation related gene expression in the absence or presence of the cultured macrophages with high LDL-cholesterol and oxidized LDL-cholesterol. ③To investigate the cholesterol uptake from the mother to macrophages in the placenta, the pregnant female mice will be injected with APOB-labeled particle ([^125^I]-LDL) or LDL-cholesterol ([^3^H]-CE-LDL) *via* tail veins at 17.5 days post coitum (dpc). Macrophages in the placenta will be isolated at 30 min, 1, 2, and 4 h after injection. Radioactivity in the homogenate of macrophages will be measured by γ-counting.

## Data Availability Statement

The original contributions presented in the study are included in the article/supplementary material, further inquiries can be directed to the corresponding author/s.

## Author Contributions

JinC and JiaC conceived the idea. JinC and FL wrote the manuscript. JiaC, LH, and JinC collected and read the literature. All authors read and approved the final manuscript. All authors contributed to the article and approved the submitted version.

## Funding

This work was supported by the grants from Hunan Provincial Natural Science Foundation of China (2022JJ40675 to JinC and 2021JJ40852 to FL), the National Natural Science Foundation of China (82100495 to FL), and Scientific Research Project of Hunan Provincial Health Commission (202203014009 to FL).

## Conflict of Interest

The authors declare that the research was conducted in the absence of any commercial or financial relationships that could be construed as a potential conflict of interest.

## Publisher's Note

All claims expressed in this article are solely those of the authors and do not necessarily represent those of their affiliated organizations, or those of the publisher, the editors and the reviewers. Any product that may be evaluated in this article, or claim that may be made by its manufacturer, is not guaranteed or endorsed by the publisher.
